# Synthesis of Novel Peptide Dendrimers PDL-GB2 and PDL-G2

**DOI:** 10.1155/2015/907859

**Published:** 2015-03-22

**Authors:** Lin Yunzhu, Weng Lingling, Qi Qingrong

**Affiliations:** ^1^Department of Pharmacy, Evidence-Based Pharmacy Center, West China Second University Hospital, Sichuan University, Chengdu 610041, China; ^2^Key Laboratory of Birth Defects and Related Diseases of Women and Children, Sichuan University, Ministry of Education, Chengdu 610041, China; ^3^Key Laboratory of Drug-Targeting and Drug-Delivery Systems of the Ministry of Education, West China School of Pharmacy, Sichuan University, Chengdu, Sichuan 610041, China

## Abstract

Peptide dendrimers are a novel type of macromolecules with precise structure, which can be used as drug target vector and controlled-release carrier. So it is valuable to study. In this paper, novel peptide dendrimers PDL-GB2 and PDL-G2 were prepared according to divergent procedure with four-orientation molecule as the core and L-lysine as the branch unit. And the structures were identified by ^1^HNMR, ^13^CNMR, MS, and elemental analysis.

## 1. Introduction

**Figure 1:**
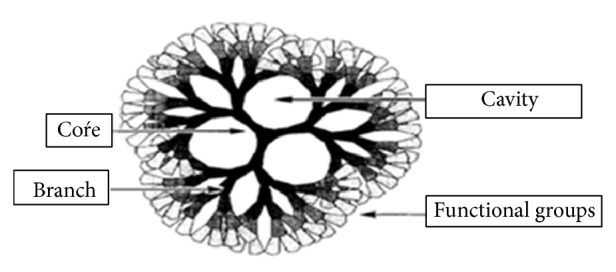
The classic dendrimer.

Dendrimers are a sort of high-molecular compound with peculiar properties which possess high symmetry in geometry morphology, precise molecular structure, and a number of functional groups (see Figure 1). Besides, the cavities and the branch length of the dendrimers can be controlled well.

Based on these features, dendrimers show a good application prospection as drug target vector and controlled-release carrier [1–6]. Some small molecules can be attached to the dendrimers according to physical inclusion or noncovalent combination. The complex expresses integral physicochemical properties. Thus the poor water solubility or the poor stability of the small molecules could be increased [7–10]. Besides this, the drug molecules can be connected to the dendrimers by covalent bond or intermolecular electrostatic attraction according to the numerous functional groups on the surface [11–13].

**Figure 2:**
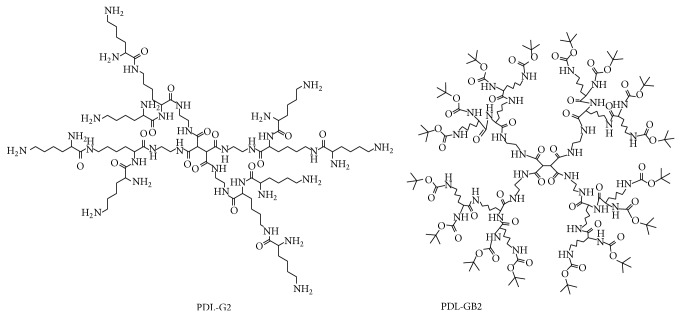
The Structures of PDL-G2 and PDL-GB2.

However, these series of studies are mostly based on polyamidoamine dendrimer (PAMAM), which was synthesized by Tomalia et al. in 1985 [14]. Recently more literatures about peptide dendrimers which could be applied in medical science were reported [15–19]. This paper described the preparation and structure confirmation of two novel peptide dendrimers PDL-GB2 and PDL-G2 (see Figure 2). The drug molecules can be encapsulated in the internal cavity of PDL-GB2 and can be linked to PDL-G2 by its surface amino groups. Both PDL-GB2 and PDL-G2 are likely to be the drug target vector and controlled-release carrier.

## 2. Materials and Methods

**Scheme 1:**
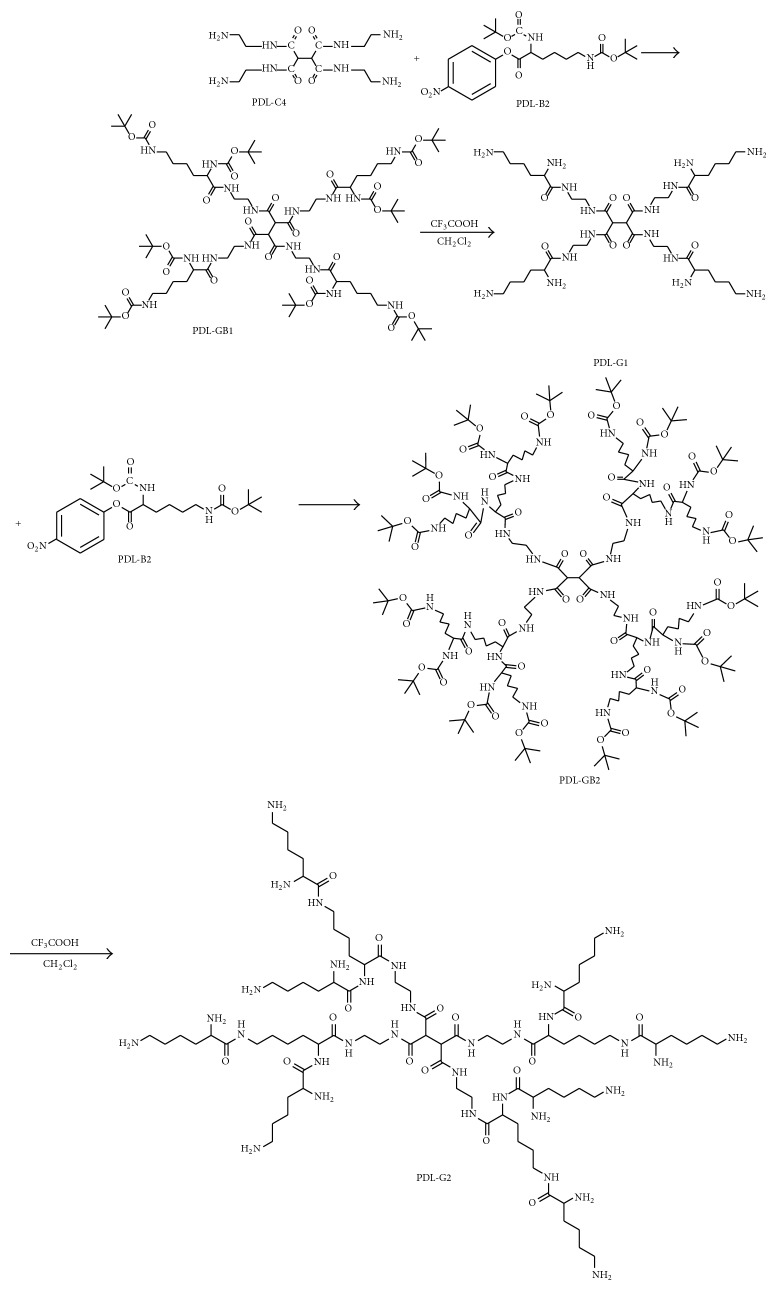
Synthesis of PDL-GB2 and PDL-G2.

The synthesis of PDL-GB2 and PDL-G2 was achieved through divergent strategy [20] shown in Scheme 1.

The starting compound PDL-C4 was reacted with PDL-B2 to produce PDL-GB1. PDL-GB1 was easily decomposed to expose four amino groups in the presence of CF_3_COOH in CH_2_Cl_2_, resulting in the colorless vitreous solid PDL-G1. In a similar way to the preparation of compound PDL-G1, PDL-G2 was synthesized. Compounds PDL-GB1, PDL-G1, PDL-GB2, and PDL-G2 have not been reported previously.

### 2.1. Major Materials and Instruments

#### 2.1.1. Reagents

All the reagents were commercially available.

#### 2.1.2. The Main Instruments

Melting point was measured through capillary tube method. Mass measurement was made by Walters Q-TOF Premier type. NMR spectra were obtained on Varian Unity INOVA NMR measurement using TMS as the internal standard. Elemental analysis was carried out on CARLO-ERBA 1106 type instrument.

### 2.2. Preparations of All the Compounds

#### 2.2.1. Synthesis of PDL-C4 (See Scheme 2)

*(1) Preparation of Diethyl 2-Bromomalonate (PDL-C1) [21]*. Diethyl malonate 160.0 g (1.0 mol) and CCl_4_ 150 mL were successively added into a 500 mL three-necked dry flask. After installation of condenser, drying tube, and constant exhaust gas absorption, three drops of Br_2_ were dropped in the mixture slowly and the radical reaction was initiated with light. When the reaction mixture faded, Br_2_ was dropped into the reaction mixture continuously. Until the consumption of Br_2,_ the mixture was refluxed for 1.5 h. After the consumption of diethyl malonate monitored by TLC (cyclohexane : acetone 1 : 1), the reaction mixture was cooled to room temperature. Then the mixture was washed with 5% Na_2_CO_3_ solution, distilled water, and dried (Na_2_SO_4_) and concentrated in vacuo (120–123°C/15 mmHg) to obtain colorless transparent liquid 204.8 g with yield 85.7%.

*(2) Preparation of Tetraethyl Ethene-1,1,2,2-tetracarboxylate (PDL-C2) [22]*. PDL-C1 120.0 g (0.5 mol), anhydrous K_2_CO_3_ 69.0 g (0.5 mol), and DMF 250 mL were added to a 500 mL three-necked dry flask and vigorously stirred at room temperature for 24 h. After the consumption of PDL-C1 monitored by TLC (cyclohexane : acetone 1 : 1), the mixture was filtered. DMF in the filtrate was removed in vacuo. And diethyl ether 100 mL was added to the residue to give a pale yellow solution. After evaporation of diethylether, the crude product was gained. And the crude product was recrystallized from ethanol to obtain white crystal 52.8 g with yield of 54.2%, mp 55–57°C.

*(3) Preparation of Tetraethyl Ethane-1,1,2,2-tetracarboxylate (PDL-C3) [23]*. 5% Pd/C 0.1 g, PDL-C2 1.0 g (3.0 mmol), and ethanol 25 mL were stirred at 40°C for 8 h under hydrogen. After the consumption of PDL-C2 monitored by TLC (cyclohexane : acetone 2 : 1), the reaction was filtered to give a colorless filtrate. The filtrate was concentrated to obtain white needle crystal 0.97 g with yield of 96.3%, mp 69–71°C.

*(4) Preparation of N,N*′*,N*′′*,N*′′′*-Tetrakis(2-aminoethyl)ethane-1,1,2,2-tetracarboxamide Hydrochloride (PDL-C4)*. PDL-C3 15.0 g (0.047 mol), absolute ethanol 50 mL, and ethylenediamine 80 mL were stirred at room temperature for 5 h. The mixture was filtered, washed with ethanol and ether, and dried to give white powder 16.8 g with yield of 81.6%. Then the white powder was dispersed in 60 mL methanol and treated with concentrated hydrochloric to adjust the system to pH2, and a lot of white granular solid was precipitated, filtered, and dried in vacuo to obtain white solid 12.0 g with yield of 51.4%.

#### 2.2.2. Preparation of 4-Nitrophenyl 2,6-Bis((tert-butoxycarbonyl)amino)hexanoate (PDL-B2) [24]

L-Lysine hydrochloride 4.0 g (21.9 mmol) was dissolved in 48 mL 1 N NaOH aqueous solution; then *t*-butanol 15 mL was added. The mixture was cooled below 15°C, and (BOC)_2_O 10.3 g (47.1 mmol) in *t*-butanol 8 mL was dropped. When (BOC)_2_O was completed, the mixture was stirred at room temperature for 8 h. Then the reaction mixture was concentrated and extracted with *n*-pentane (10 mL × 3). The aqueous phase was washed with 1 N KHSO_4_ to adjust pH2 and extract with ethyl acetate (20 mL × 3). Ethyl acetate phases were combined, washed with distilled water (5 mL × 3), dried (Na_2_SO_4_), and concentrated to give colorless oil 6.08 g with yield of 80.1%.

The colorless oil 6.01 g (17.3 mmol) and *p*-nitrophenol 2.65 g (19.0 mmol) were dissolved in 20 mL ethyl acetate and cooled to 0°C. DCC 3.93 g (19.0 mmol) dissolved in 10 mL ethyl acetate was added dropwise to the reaction at 0°C. Then the mixture was stirred at room temperature for 8 h. 1 mL glacial acetic acid was added and the mixture was stirred at room temperature for 30 min. Then the mixture was filtered and the filtrate was concentrated to give white solid, which was recrystallized from ethanol to obtain 6.85 g of white solid, mp 116–118°C. The yield was 84.6%.

#### 2.2.3. Preparation of N,N′,N′′,N′′′-Tetrakis (*α*-aminoethyl)-1,1,2,2-ethane Tetracarboxylic Acid Amide (4): (L-lysine): N N′-di-t-Butoxycarbonyl-L-lysine (PDL-GB1)

PDL-C4 1.40 g (2.7 mmol), triethylamine 1.10 g, PDL-B2 7.50 g (16.0 mmol), and DMSO 40 mL were stirred at room temperature for 72 h and additional triethylamine was added dropwise in portions during the reaction in order to maintain pH8-9. After 72 h, the reaction solution was poured into 500 mL distilled water, and the precipitated solid was stirred at room temperature for 4 h. Then the solid was filtered, washed with water, and dried to give a yellow solid. The yellow solid was washed with diethyl ether to obtain a white solid 4.00 g with yield of 89%, mp > 200°C.

#### 2.2.4. Preparation of N,N′,N′′,N′′′–Tetrakis(2-(2,6-diaminohexanamido)ethyl)ethane-1,1,2,2-tetracarboxamide (PDL-G1)

PDL-GB1 0.30 g (0.18 mmol) was added into a mixed solution involved trifluoroacetic acid 8 mL and dichloromethane 8 mL. The reaction was stirred at room temperature for 2 h and concentrated in vacuo to give colorless hard glass.

#### 2.2.5. Preparation of N,N′,N′′,N′′′-Tetrakis (*α*-amino-ethyl)-amide Four Ethane (4): (L-lysine): N,N′-di-t-Butoxycarbonyl-L-lysine (PDL-GB2) 

PDL-GB1 0.30 g (0.18 mmol) was added into a mixed solution involved trifluoroacetic acid 8 mL and dichloromethane 8 mL. The reaction was stirred at room temperature for 2 h and concentrated in vacuo to give colorless hard glass. Then this product was dissolved in 15 mL DMSO and adjusted pH to 8-9 using triethylamine. PDL-B2 1.00 g (2.1 mmol) which was dissolved in 5 mL DMSO was added into the mixture and was stirred at room temperature. During the reaction, triethylamine was needed to add dropwise in portions to maintain pH 8-9. The reaction mixture should be poured into 250 mL distilled water till the reaction had been carried out for 7 h at room temperature. Then the mixture was stirred at room temperature for 6 h, filtered, and washed repeatedly with water and dried to get brown solid. This crude product was washed with diethyl ether and dried to give slightly yellow solid 0.60 g with yield of 96.8%, mp > 200°C.

#### 2.2.6. Preparation of N,N′,N′′,N′′′-Tetrakis(2-(2,6-bis(2,6-diaminohexanamido)hexanamido)ethyl)ethane-1,1,2,2-tetracarboxamide (PDL-G2)

PDL-GB2 0.30 g (0.085 mmol) was added into a mixed solution of trifluoroacetic acid 8 mL and dichloromethane 8 mL. The reaction mixture was stirred at room temperature for 4 h. Then the solvent was removed in vacuo to obtain colorless transparent rigid glass.

## 3. Results and Discussion

The structures of all the intermediates and target compounds described above have been confirmed by ^1^HNMR, ^13^CNMR, MS, and elemental analysis.

 PDL-C2: ^1^HNMR (400 MHz, CDCl_3_): *δ* 4.28 (q, *J* = 7.2 Hz, 8H), 1.27 (t, *J* = 7.2 Hz, 12H); ^13^CNMR (100 MHz, CDCl_3_): *δ* 162.3, 135.3, 62.5, 12.7.
 PDL-C4: ^1^HNMR (400 MHz, DMSO-d6): *δ* 7.74 (s, 4H), 3.97 (t, *J* = 3.2 Hz, 2H), 2.99 (m, 8H), 2.30 (m, 8H); ^13^CNMR (100 MHz, D_2_O): *δ* 172.3, 55.8, 41.7, 40.1.
 PDL-B2: ^1^HNMR (400 MHz, DMSO-d6): *δ* 8.20 (m, 2H), 7.28 (m, 2H), 5.29 (m, 1H), 4.62 (m, 1H), 4.46 (m, 1H), 3.16 (m, 2H), 1.46 (m, 24H).
 PDL-GB1: ^1^HNMR (400 MHz, DMSO-d6): *δ* 7.80 (m, 8H), 6.74 (s, 4H), 6.73 (s, 4H), 3.90 (s, 2H), 3.81 (m, 4H), 3.04 (s, 16H), 2.87 (s, 8H), 1.20–1.40 (m, 96H); ^13^CNMR (100 MHz, DMSO-d6): *δ* 172.5, 167.7, 155.7, 155.5, 78.1, 77.5, 54.5, 52.4, 31.7, 29.4, 26.4, 23.0; *m*/*z*/[M+H]^+^ calcd for C_78_H_142_N_16_O_24_ 1688.0549, Found 1688.0444. Anal calcd for C_78_H_142_N_16_O_24_: C: 55.50, H: 8.48, N: 13.78, O: 22.75; Found: C: 54.96, H: 8. 67, N: 12.98, O: 23.39.
 PDL-G1: ^1^HNMR (400 MHz, DMSO-d6): *δ* 8.55 (m, 4H), 8.32 (m, 16H), 8.08 (m, 4H), 3.93 (s, 2H), 3.71 (s, 4H), 3.12 (m, 16H), 2.76 (m, 8H), 1.80 (m, 8H), 1.54 (m, 8H), 1.24 (m, 8H); ^13^CNMR (100 MHz, DMSO-d6): *δ* 168.7, 167.8, 125.5, 123.0, 52.3, 30.5, 26.2, 21.3.
 PDL-GB2: ^1^HNMR (400 MHz, DMSO-d6): *δ* 7.90 (m, 16H), 6.90 (m, 4H), 6.74 (m, 12H), 4.17 (s, 2H), 3.82 (s, 12H), 3.03 (m, 16H), 2.87 (m, 24H), 1.2–1.6 (m, 216H); ^13^CNMR (100 MHz, DMSO-d6): *δ* 172.2, 171.8, 167.7, 155.8, 155.5, 78.2, 77.8, 54.8, 54.5, 52.4, 32.1, 31.6, 29.4, 29.0, 28.5, 28.4, 23.1, 22.9; *m*/*z*/[M+2Na]^2+^ calcd for C_166_H_302_N_32_O_48_ 1779.1087, Found 1779.11. Anal calcd for C_166_H_302_N_32_O_48_: C: 56.73, H: 8.66, N: 12.75, O: 21.85. Found C: 55.27, H: 8. 69, N: 12.02, O: 24.02. The error between measured value and theoretical value was reasonable for dendrimer [25].
 PDL-G2: ^1^HNMR (400 MHz, DMSO-d6): *δ* 8.59 (m, 4H), 8.48 (m, 4H), 8.31 (m, 16H), 8.06–8.18 (m, 16H), 7.86 (m, 16H), 4.19 (s, 2H), 3.84 (s, 4H), 3.70 (m, 8H), 3.08 (m, 16H), 2.76 (m, 24H), 1.71 (m, 48H), 1.55 (m, 24H); ^13^CNMR (100 MHz, DMSO-d6): *δ* 172.1, 169.2, 166.7, 166.3, 126.0, 123.5, 53.7, 52.78, 52.76, 31.8, 31.0, 30.0, 29.1, 27.4, 27.0, 23.4, 21.7.

**Figure 3:**
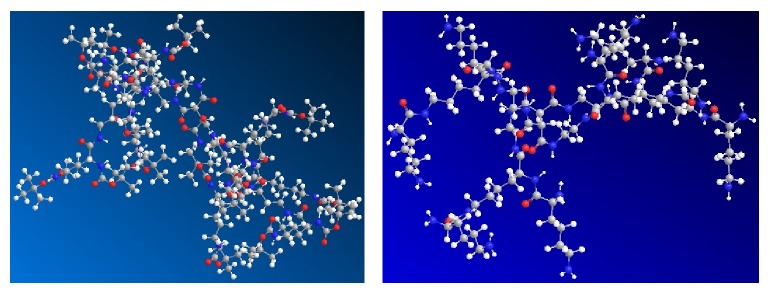
The computer simulation spacial structures of PDL-GB2 and PDL-G2. Blue: N atom; red: O atom; gray: C atom; white: H atom.

Dendrimers are generally made up of the initial core, branches, and terminal functional groups. The core of PDL-GB2 and PDL-G2 mentioned in this paper was the same four-orientation symmetric structure, which was used in peptide dendrimers for the first time. The number of branch unit was appropriate helping to generate the growth in subsequent dendrimers. The branches could change the structures of cavities and the ability of parceling drug molecules of dendrimers. In this work, natural amino acid L-lysine was chosen as the branch unit, and the free amino of L-lysine was expected to be the functional groups on the dendrimer surface. Meanwhile, compounds PDL-GB2 and PDL-G2 were expected to possess the cavities in their structures. To test this idea, three-dimensional molecular model of compounds PDL-GB2 and PDL-G2 were simulated using ChemBio 3D software (Figure 3).

The pictures showed that the spatial structure of PDL-GB2 was similar to “dendritic box” with cavities in the molecule, which meant drug molecule could be carried in the cavities. While compound PDL-GB2 got in the acidic environment in body, BOC groups on the surface of PDL-GB2 could be removed to help drug molecules release easily. Amino groups which are located on the surface of PDL-G2 were conducive to connect some drug molecules and could release these drugs in suitable environment. These two molecules were all likely to achieve the purpose of controlled-release as well as targeted delivery.

## 4. Conclusion

Novel peptide dendrimers PDL-GB2 and PDL-G2 were synthesized using four-orientation core PDL-C4 and L-lysine branch unit. The spatial structures of PDL-GB2 and PDL-G2 simulated by computer showed that the two molecules were likely to be targeted drug delivery and controlled-release materials. Meanwhile, all the dendrimers synthesized in this work were all connected by amide bonds, which could degrade easily in body to avoid accumulation. Further studies of its biological activities are in progress.

## Figures and Tables

**Scheme 2:**
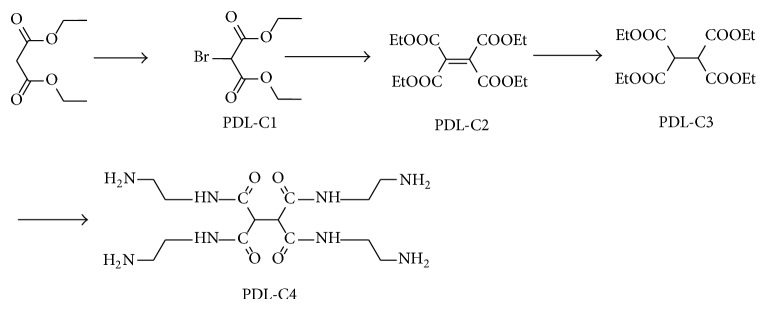
Synthesis of PDL-C4.
